# A review of combined neuromodulation and physical therapy interventions for enhanced neurorehabilitation

**DOI:** 10.3389/fnhum.2023.1151218

**Published:** 2023-07-21

**Authors:** Alexandra Evancho, William J. Tyler, Keith McGregor

**Affiliations:** ^1^Department of Physical Therapy, School of Health Professions, University of Alabama at Birmingham, Birmingham, AL, United States; ^2^Department of Biomedical Engineering, Heersink School of Medicine, University of Alabama at Birmingham, Birmingham, AL, United States; ^3^Department of Physical Medicine and Rehabilitation, Heersink School of Medicine, University of Alabama at Birmingham, Birmingham, AL, United States; ^4^Department of Clinical and Diagnostic Studies, School of Health Professions, University of Alabama at Birmingham, Birmingham, AL, United States

**Keywords:** neuromodulation, neuroplasticity, rehabilitation, vagus nerve stimulation, transcranial magnetic stimulation, peripheral nerve stimulation

## Abstract

Rehabilitation approaches for individuals with neurologic conditions have increasingly shifted toward promoting neuroplasticity for enhanced recovery and restoration of function. This review focuses on exercise strategies and non-invasive neuromodulation techniques that target neuroplasticity, including transcranial magnetic stimulation (TMS), vagus nerve stimulation (VNS), and peripheral nerve stimulation (PNS). We have chosen to focus on non-invasive neuromodulation techniques due to their greater potential for integration into routine clinical practice. We explore and discuss the application of these interventional strategies in four neurological conditions that are frequently encountered in rehabilitation settings: Parkinson’s Disease (PD), Traumatic Brain Injury (TBI), stroke, and Spinal Cord Injury (SCI). Additionally, we discuss the potential benefits of combining non-invasive neuromodulation with rehabilitation, which has shown promise in accelerating recovery. Our review identifies studies that demonstrate enhanced recovery through combined exercise and non-invasive neuromodulation in the selected patient populations. We primarily focus on the motor aspects of rehabilitation, but also briefly address non-motor impacts of these conditions. Additionally, we identify the gaps in current literature and barriers to implementation of combined approaches into clinical practice. We highlight areas needing further research and suggest avenues for future investigation, aiming to enhance the personalization of the unique neuroplastic responses associated with each condition. This review serves as a resource for rehabilitation professionals and researchers seeking a comprehensive understanding of neuroplastic exercise interventions and non-invasive neuromodulation techniques tailored for specific diseases and diagnoses.

## Introduction

**FIGURE 1:**
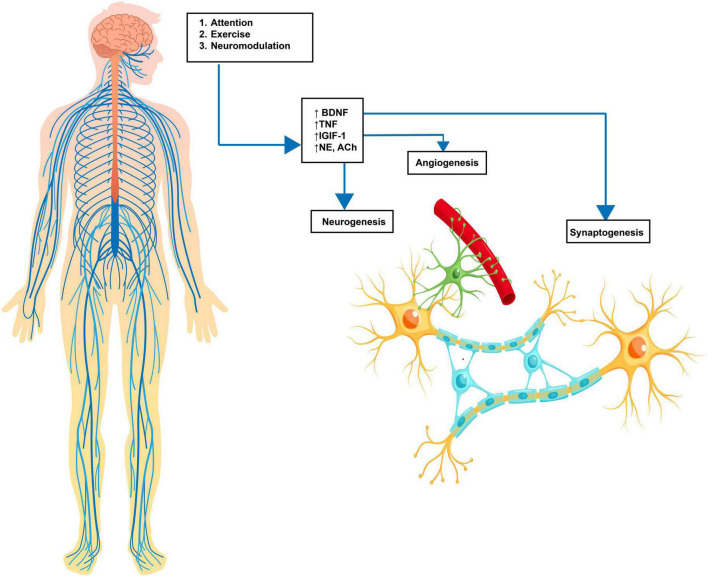
Neuroplasticity cascade. Enviromental input via learning, exercise or neuromodulation causes an increase in molecular substrates, including BDNF (brain-derived neurotrophic Factor), TNF (tumor necrosis factor), NE (norepinephrine), DA (dopamine), and 5-HT (serotonin). These molecular changes lead to neuroplastic cellular changes, including angiogenesis (new blood supply), neurogenesis (new neuronal growth), and synaptogenesis (increased synaptic connectivity). The result is an increased strength of neural pathways. Source: Biorender.com.

Neuroplasticity refers to the brain’s unique capacity to reorganize, modify, and adjust. It encompasses the central nervous system’s ability to forge, reinforce, and restructure neural connections in response to changes in sensory inputs or motor demands. Neuroplasticity plays a crucial role in the acquisition of new skills as well as in the recovery and rehabilitation processes following the diagnosis of various neurological conditions. This involves a complex interplay of coordinated neurotransmitter release, including acetylcholine (ACh), norepinephrine (NE), epinephrine (Epi), and dopamine (DA). The release of these neurotransmitters induces changes in both white and gray matter through neurogenesis, synaptogenesis, angiogenesis, and gliogenesis (Zatorre et al., 2012), thereby creating stronger and more efficient connections within the relevant neural pathways (Figure 1). These changes enable more efficient information translation through neural circuits and aid in successful task completion. The effectiveness of both exercise and neuromodulation can be attributed to their ability to promote neuroplastic change.

Understanding how natural and induced neuroplastic changes can occur in the brain is vital for the development of effective rehabilitative treatments involving exercise and neuromodulation. For example, neuroplasticity-based approaches may exhibit differing impacts, depending on whether the individual has experienced a neurological insult or is living with a neurodegenerative disease. Following neurological insults such as stroke, traumatic brain injury (TBI), and spinal cord injury (SCI), neuroplastic changes occur naturally to compensate for new structural and functional deficits. This compensation is achieved through the rewiring of neural pathways and significant cortical alterations in brain function (Nudo, 2007). Therefore, an individual who has experienced a neurological insult may display a heightened state of neuroplasticity following the event as the brain actively works to restore lost function. In contrast, individuals with neurodegenerative diseases experience a gradual and progressive loss of neuronal function and structure. In this case, the goal of a neuroplasticity-based approach is to slow this decline by stimulating the brain’s inherent plasticity to compensate for the loss of function over time. Ultimately, optimizing patient outcomes through a neuroplasticity-based approach hinges upon a deep understanding of the neuroplastic changes that occur in distinct pathological conditions, and how we can strategically manipulate the human nervous system through exercise and neuromodulation to enhance neuroplasticity.

In this review, we explore the intersection of neuromodulation and rehabilitation, investigating how these combined approaches shape neuroplastic changes across a range of neurologic diagnoses. Our primary focus is on the motor aspects of these conditions, as the enhancement of motor function is the primary aim of physical therapy in neurorehabilitation. However, we also acknowledge that these condition also impact non-motor aspects, and we touch on non-motor aspects throughout the manuscript. We have selected to discuss both neurodegenerative disease and neurological insults, considering their distinct mechanisms of action that could affect their response to neuroplasticity. Given the vast spectrum of research available, the constraints of space, and individual variations in treatment responses, we will concentrate on how exercise and neuromodulation promote neuroplasticity within a selection of neurological diagnoses commonly encountered in the rehabilitation setting. The conditions we have chosen to focus on include Parkinson’s Disease (PD), Traumatic Brain Injury (TBI), Stroke, and Spinal Cord Injury (SCI). Subsequently, we delve into three non-invasive neuromodulation techniques – Transcranial Magnetic Stimulation (TMS), Vagus Nerve Stimulation (VNS), and Peripheral Nerve Stimulation (PNS). These techniques have been chosen due to the available evidence highlighting these modalities’ capacity to induce neuroplastic changes. Additionally, their non-invasive nature amplifies their relevance in a clinical context, indicating a higher likelihood of integration into rehabilitation settings. We will also briefly touch upon emerging technologies such as transcranial direct current stimulation (tDCS) and focused ultrasound (FUS), acknowledging their potential and indicating areas where further research is needed for their safe and effective implementation in clinical practice.

Our primary objective is to offer a comprehensive overview of the existing literature concerning the application of combined exercise and neuromodulation in rehabilitation for selected diagnoses. To achieve this, we start by examining the role of exercise in enhancing neuroplasticity across the selected conditions. We then transition into a discussion of neuromodulation approaches, including the latest research findings within each diagnosis. Within these sections, we present studies that have effectively integrated exercise and neurostimulation for enhanced outcomes, advocating for a combined approach of neuromodulation and physical therapy. Finally, we propose specific, innovative interventions that merge exercise and neurostimulation for each diagnosis discussed, paving the way for future research in this promising field. By understanding the unique neural targets, optimal intervention timing and parameters, and the appropriate type and intensity of treatment protocols for each patient, we aim to establish a foundational framework for integrating exercise and neurostimulation approaches in the rehabilitation setting. The suggested interventions offered in this article promote potential research directions, emphasizing careful intervention selection tailored to each patient’s diagnosis, while considering each intervention’s unique neuroplastic effects. By adopting this neuroscience-guided approach, we aim to ultimately enhance patient outcomes and contribute to the advancement of the field of neurorehabilitation.

## Exercise and neuroplasticity

While the positive effects of exercise on overall health have been well established (Powell and Paffenbarger, 1985; Booth et al., 2002) it is only during the last decade that researchers have delved more deeply into the effects of exercise on brain health (van Praag, 2009). Exercise, specifically aerobic exercise, offers a wealth of benefits for brain health, including enhancing neuroplasticity by creating a neural environment that is primed for change. One of the pathways through which exercise induces neuroplasticity is the production of brain-derived neurotrophic factor (BDNF) (Murer et al., 2001), a protein that promotes the growth and survival of neurons. Exercise stimulates the release of BDNF, setting off a chain of events leading to structural and functional transformations within the brain (Cotman et al., 2007; Hamilton and Rhodes, 2015). For example, increased levels of BDNF activate tropomyosinrelated receptor kinase B (TrkB), which influences neuronal dendritic spine growth (synaptogenesis) (Guo et al., 2018), ultimately increasing post-synaptic drive to the motor neuron and improving nervous system communication. This increase in BDNF with exercise has been observed in both animal (Neeper et al., 1996) and human studies (Rasmussen et al., 2009), serving as an important mechanism underlying exercise-induced neuroplasticity and cognitive enhancement (Berchtold et al., 2005; Intlekofer et al., 2013). In addition to BDNF production, exercise also elevates insulin-like growth factor-1 (IGF-1), which, like BDNF, facilitates exercise-induced growth of blood vessels (angiogenesis) (Ding et al., 2006) and the formation of new neurons (neurogenesis) (Lopez-Lopez et al., 2004). Additionally, IGF-1 stimulates BDNF upregulation (Carro et al., 2001; Ding et al., 2006), further contributing to enhanced neural function and cognitive performance. Another growth factor contributing to the beneficial effects of exercise is vascular endothelial growth factor (VEGF), which is linked to the proliferation of neurons and the growth of blood vessels (Fabel et al., 2003). Overall, these changes at a cellular and molecular level drive changes in brain structure (both white and gray matter) and function, resulting in an increased efficiency of neural activation and communication, ultimately changing cognitive and motor performance (El-Sayes et al., 2019). It is worth noting that while these systemic effects create a favorable environment for neuroplasticity, the specific skills acquired through these changes may depend on other factors and interactions.

## Implications for exercise-based therapies across neurological disorders and injury

### Parkinson’s disease

Parkinson’s disease is a degenerative neurological condition that gradually damages dopamineproducing neurons in two regions of the brain, namely the substantia nigra pars compacta (SNc) and ventral tegmental area (VTA), leading to dopamine deficiency. The disease presents with both motor (bradykinesia, hypokinesia, rigidity, and tremor) and non-motor (depression, cognitive impairment, apathy, visual impairments, fatigue, and insomnia) symptoms, all of which negatively impact quality of life. An expanding body of research highlights the significance of physical exercise in managing Parkinson’s Disease symptoms through enhanced neuroplasticity (Johansson et al., 2020). In a comprehensive 2020 review article, Johanssen et al. conducted a meta-analysis and synthesis of exercise studies in humans with PD that looked at neuroplastic markers. They determined that various forms of physical exercise may lead to changes in a range of markers of neuroplasticity including increases in blood/serum BDNF and BDNF-TrkB signaling. Additionally, they evaluated studies that measured changes in brain structure and function, finding increased dopamine transmission, increased corticomotor excitability, weakened overactive indirect striatal pathway DA-D2R expression, and changes in gray matter volume with exercise (Mackay et al., 2017). Of note is that exercise intensity is a crucial factor in eliciting neuroplastic changes, with most studies recommending moderate-high intensity exercise interventions. The concept proposed is not merely to achieve a cardiovascular or metabolic challenge, but to engage the brain with specific, complex and coordinated activity that demands a higher degree of cortical involvement. LSVT BIG, a research-based exercise protocol developed from Lee Silverman’s LSVT LOUD speech therapy program, is a PD specific intervention designed to improve function and slow progression of motor symptoms (Fox et al., 2012; Isaacson et al., 2018; Flood et al., 2020; Fleming Walsh et al., 2022). LSVT BIG incorporates principles aligned with the literature that identifies fundamental components of exercise that enhance neuroplasticity and promote brain reorganization, including specificity, intensity, repetition, and salience of treatment. An updated variant of this program, known as Parkinson’s Wellness Recovery (PWR), could potentially provide both learning and cardiovascular benefits. Such moderate to high-intensity exercise protocols, including LSVT BIG or PWR, should be employed when treating individuals with PD to stimulate neuroplasticity and promote optimal functional recovery. For more information, refer to the 2022 clinical practice guideline by Osborne et al. published in the Journal of Physical Therapy for a comprehensive approach (Osborne et al., 2022).

### Stroke

Stroke is one of the most prevalent neurological conditions worldwide, resulting in physical impairments including hemiparesis (weakness on one side of the body) and/or hemiplegia (paralysis on one side of the body). Following stroke, patients typically experience the most significant improvements in physical function within the first three months, and then progress plateaus (Jørgensen et al., 1995a,b). Despite this initial spontaneous remodeling, these changes are often insufficient in producing functional recovery (Xing and Bai, 2020). However, patients that engage in physical rehabilitation demonstrate greater improvement in functional skills compared to those who do not (Pollock et al., 2014). Effective post-stroke rehabilitation utilizes key principles of neuroplasticity to restore motor function (Dobkin and Carmichael, 2016). Constraint Induced Movement Therapy (CIMT), a series of interventions that force patients to utilize their affected limb to perform salient and repetitive tasks, can increase dendritic projections and reestablish axonal connections between hemispheres (Nesin et al., 2019). This intervention results in brain reorganization and changes in brain function (Mark et al., 2006), including expanded representation of the affected limb in the motor cortex, improving limb use for individuals with chronic stroke hemiparesis (Taub et al., 1993). Other stroke rehabilitation interventions, including aerobic exercise and general task-specific training, have identified increased production of BDNF as a key facilitator of motor learning during neuroplastic rehabilitation (Mang et al., 2013). Overall, exercise facilitates neuroplasticity post-stroke in multiple ways, including increasing synaptic plasticity, dendritic and axonal growth and function improving interhemispheric connection, promoting neural regeneration and organization, and increasing strength of surviving brain areas (Xing and Bai, 2020). Therefore, guided by evidence-based practice (Taub et al., 2002; Chen et al., 2019), clinicians can choose from an array of exercise interventions known to induce neuroplastic changes, tailoring the selection to the specific needs and circumstances of the individual recovering from stroke.

### Traumatic brain injury

Traumatic brain injury (TBI) is a broad category encompassing a range of injury severities, mechanisms, and presentations. It is primarily characterized by brain damage occurring after birth, independent of congenital or developmental conditions (National Institute of Neurological Disorders and Stroke, 2022). The heterogeneity of TBI can cause a wide range of impairments, and TBI classifications (mild, moderate, severe) each present unique characteristics and therapeutic needs. Broadly speaking, almost 30% of patients experience balance issues (Basford et al., 2003) and motor functional limitations including gait abnormalities (Marshall et al., 2007) after TBI. Disrupted vestibular function is also often comorbid with TBI (Mucha et al., 2018; Marcus et al., 2019), and vestibular rehabilitation is commonly prescribed to treat vestibular dysfunction. Additionally, neuroinflammation is a significant secondary consequence following traumatic brain injury (TBI), playing a substantial role in subsequent cell death (Kumar and Loane, 2012). This inflammatory response is initiated immediately following the traumatic event and can persist for an extended period, potentially up to 17 years post-TBI (Johnson et al., 2013). While this response initially aims to repair damaged cells and protect the brain from potential pathogen invasion, excessive and persistent inflammation can have harmful effects (Wofford et al., 2019). In animal models, exercise after TBI can counteract neuroinflammation and promote neuroprotection; however, only after a period of delay after injury (Piao et al., 2013). Excessive exertion with premature exercise could interrupt the natural restorative processes triggered after TBI (Griesbach, 2011), and both animal and human studies have revealed that early initiation of exercise following TBI might inhibit neuroplasticity and deteriorate outcomes (Griesbach, 2011). Accordingly, preliminary efforts have been made to develop protocols that consider these conflicting effects of exercise on neurorecovery. For example, the Buffalo Concussion Treadmill Test was developed for sports-related concussion, which is a type of mild TBI. This assessment acts as a return-to activity assessment (Leddy and Willer, 2013), which allows clinicians to record the individuals threshold of symptom exacerbation. The results of this assessment can be used to safely prescribe a progressive aerobic exercise program (Leddy et al., 2018). Overall, if initiated in the appropriate time window, postinjury exercise can enhance functional recovery, with improvements in motor performance, spatial learning and memory tasks linked to increases in exercise-induced BDNF after TBI (Griesbach et al., 2004). Additional research evaluating specific exercise protocols targeting induction of neuroplasticity is needed to fully understand the mechanisms by which exercise may promote adaptation in individuals with mild, moderate, and severe TBI. These efforts will help inform the optimal timing, intensity, and duration of training for these individuals.

### Spinal cord injury

Spinal cord injury (SCI) occurs when the spinal cord is damaged by trauma, disease, or degeneration. This can cause partial or complete loss of sensory or motor function in the arms, legs, or body (WHO, 2022). Physical exercise has been shown to have positive effects at the cellular and molecular level (Fouad and Tetzlaff, 2012), leading to the restoration of both sensory and motor function (Hutchinson et al., 2004; Sandrow-Feinberg and Houlé, 2015). For example, exercise increases synaptic plasticity after SCI by increasing the production of neurotrophic factors (Vaynman et al., 2003), which can be found in higher concentrations in spinal and muscle tissue after physical activity (Gómez-Pinilla et al., 2002; Ying et al., 2005; Côté et al., 2011). Additionally, exercise has been shown to decrease inflammation around the injury site (Sandrow-Feinberg et al., 2009). These neuroplastic effects have been linked to improved global motor function (Bilchak et al., 2021), with both treadmill (Wang et al., 2015) and bike training (Ganzer et al., 2018a) shown to increase dendritic density and total neurite length compared to sedentary controls. The clinician, however, must consider the type of spinal cord injury when prescribing neuroplastic exercise interventions. In complete SCI, there is a total loss of all motor and sensory function below the level of injury (Wilson et al., 2012). In incomplete SCI, some function remains below the primary level of injury, and individuals with this type of SCI may show faster and more significant improvements due to spared neural circuits (Little et al., 1999). Additionally, some patients with high-level SCI may not be able to tolerate the intensity of exercise required to induce neuroplastic changes due to compromised diaphragmatic function (Zimmer et al., 2007). Therefore, the type and intensity of exercise prescribed for individuals with SCI should be tailored to the individual’s specific needs and abilities.

## Neuromodulation and neuroplasticity

As defined by the International Neuromodulation Society, neuromodulation is an external alteration of nerve activity through delivery of a distinct stimulus, such as a magnetic field or electric current (International Neuromodulation Society, 2023). This technique is increasingly being studied regarding its ability to modulate neuroplastic changes in the brain (Danilov and Paltin, 2018). By altering the nervous system’s electrical activity, neuromodulation can lead to changes in brain structure or function. In the following sections, we discuss three specific non-invasive neuromodulation techniques including transcranial magnetic stimulation (TMS), peripheral nerve stimulation (PNS), and vagus nerve stimulation (VNS), and their applications in neurologic diagnoses including PD, Stroke, TBI, and SCI, which are the conditions we focus on in this review. This section aims to combine this information with the previously discussed disease-specific exercise considerations, enabling a comprehensive understanding of how to optimize the combination of neuromodulation and exercise. The goal is to outline a path toward the personalization of interventions, tailoring the treatments to each patient’s unique needs.

## Transcranial magnetic stimulation

### Overview

**FIGURE 2:**
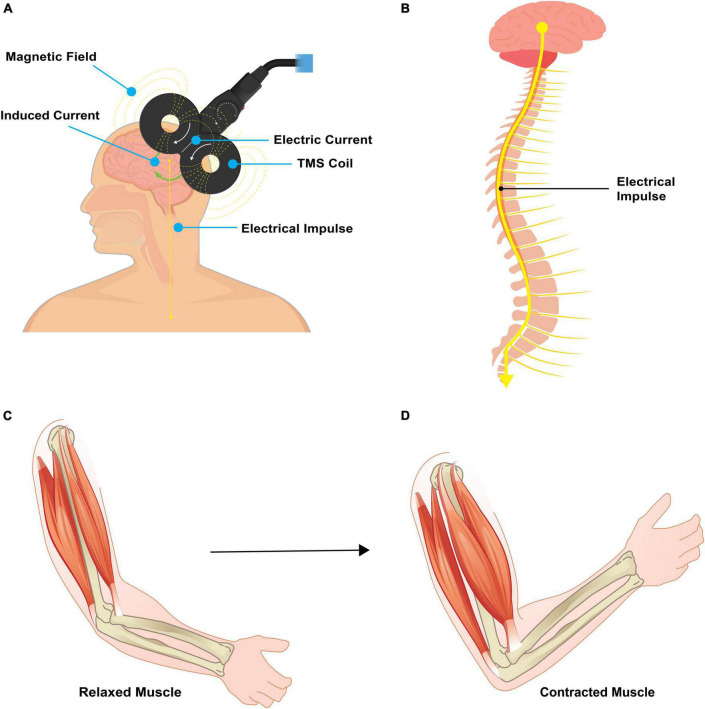
Pictorial representation of TMS. **(A)** Transcranial magnetic stimulation involves placement of a magnet coil over the scalp to induce an electric field. This electric field passes through the scalp and skull to act directly on the targeted brain area. **(B)** When applied to the primary motor cortex, an electrical impulse travels down the corticospinal tract and results in a targeted muscle contraction. **(C,D)** A relaxed muscle will contract with targeted TMS. Source: Biorender.com.

Transcranial Magnetic Stimulation (TMS) is a non-invasive neuromodulation method that utilizes a strategically placed wire coil over the scalp to induce a magnetic field. This approach generates an electric current penetrating the skull, provoking neuronal depolarization in the targeted cortical region. TMS is an enticing neuromodulation strategy due to its non-surgical application, ability to focus on precise brain regions, and capacity to incite motor contractions (Figure 2). TMS serves investigative and therapeutic roles, fostering the exploration of human brain function and promoting neuroplasticity non-invasively (O’Malley et al., 2006). When employed therapeutically, the objective of TMS is to modulate neural activity in the targeted cortical region (Kesikburun, 2022), often achieved via repetitive TMS pulses (rTMS). Notably, rTMS has been deemed safe with no lasting neurological, cognitive, or cardiovascular sequelae reported (George and Aston-Jones, 2010), further bolstering its appeal in neurorehabilitation.

**FIGURE 3:**
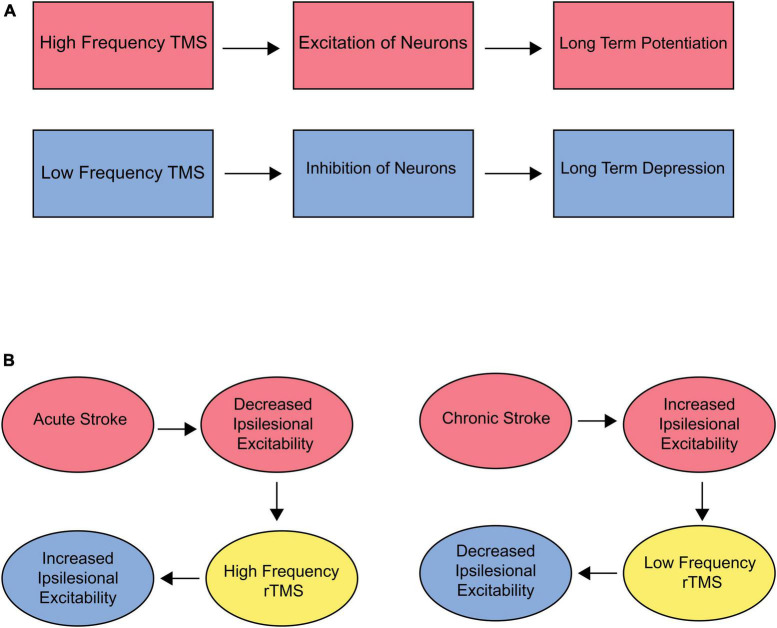
**(A)** Effect of TMS on LTP/LTD. High frequency stimulation has an overall excitatory effect on neurons, theoretically resulting in long-term potentiation (LTP). Low frequency stimulation has an inhibitory effect, resulting in long-term depression (LTD). **(B)** Target and frequency recommendations for acute and chronic stroke. Acute stroke is accompanied by decreased ipsilesional excitability. Therefore, high-frequency rTMS should be used over the ipsilesional M1 to optimize outcomes. Chronic stroke is associated with increased ipsilesional excitability. Therefore, low-frequency stimulation to downregulate the contralesional hemisphere could be beneficial.

TMS affects a number of neurophysiological processes in the brain, including long term potentiation (LTP) and long term depression (LTD) (Gersner et al., 2011). LTD and LTP are long-term changes in the strength of connections between neurons, specifically at the synapse. These changes are important for learning, skill acquisition, and memory (Abraham et al., 2019). LTP is a long-term increase in the strength of a synapse, which can be triggered by high-frequency stimulation of the synapse (Purves et al., 2001), and is responsible for Hebbian learning and formation of long-term memories. LTD is a long-term decrease in the strength of a synapse, which can be triggered by repeated low-frequency stimulation of the synapse (Gonzalez et al., 2014). This decrease in synapse strength may be important for weakening or eliminating connections between neurons that are no longer needed and is therefore considered inhibitory. Evidence suggests that TMS may be able to modulate LTD and LTP in the brain (Chervyakov et al., 2015). For example, high frequency TMS can have an excitatory effect, resulting in LTP (Tian and Izumi, 2022). On the other hand, low-frequency TMS can inhibit neuronal synapses, resulting in LTD (Casula et al., 2014) (Figure 3A). Additional neuroplastic properties of rTMS include changes in cerebral blood flow (Jung et al., 2020), increased neurotransmitter levels (Strafella et al., 2001), and modification of gene expression (Hwang et al., 2022).

## TMS implications by disease

### Parkinson’s disease

Transcranial Magnetic Stimulation (TMS), particularly repetitive TMS (rTMS), is increasingly seen as a potential therapeutic intervention for Parkinson’s Disease (PD). The growing body of research we highlight here mainly focuses on motor learning, to introduce promising implications for incorporating TMS into rehabilitation settings. Several studies have demonstrated how rTMS can mitigate motor symptoms in PD by targeting specific brain regions such as the M1, SMA, and dorsolateral prefrontal cortex with low or high-frequency protocols (Wagle Shukla et al., 2016; Goodwill et al., 2017; Yang et al., 2018). A seminal study from 2020 by Chung et al. showed that the benefits of treadmill training could be significantly amplified when the brain is primed with 1 and 25 Hz rTMS, resulting in sustained motor improvement up to three months post-intervention (Chung et al., 2020). This integration enhances activity-dependent plasticity primarily by stabilizing the consolidation process and adjusting cortical excitability. These findings demonstrate the value of pairing exercise and neuromodulation, specifically by priming. This research also notes the potential of rTMS to improve non-motor symptoms of PD, including cognition and depression (Sanches et al., 2020), further supporting the utility of TMS to address a variety of symptoms in a rehabilitative context.

Furthermore, a comprehensive 2018 meta-analysis by Yang et al. demonstrated the therapeutic potential of rTMS on the motor recovery in PD patients (Yang et al., 2018). They incorporated 23 studies with a total of 646 participants and found significant improvements in motor function in the short and long term. Importantly, high-frequency rTMS substantially impacted motor function improvement, while low frequency rTMS did not share the same effect. Among high-frequency rTMS interventions, multisession rTMS targeting bilateral M1 regions was found to be the most effective, showcasing the greatest effect size (Yang et al., 2018) and demonstrates the importance of personalized interventions to optimize patient outcomes.

### Stroke

In a healthy brain, interhemispheric communication is vital as the two brain hemispheres constantly exchange information (Gazzaniga, 2000). This balance is disrupted by stroke, leading to a complex pattern of cortical reorganization as the brain attempts to compensate for this deficit (Casula et al., 2021). The use of repetitive Transcranial Magnetic Stimulation (rTMS) has been proven to facilitate cortical reorganization in stroke patients, both in the acute and chronic phases. Doing so helps to avert abnormal reorganization and fosters functional preservation or enhancement of motor abilities (Dionísio et al., 2018).

In the subacute stroke phase, a decline in motor cortex excitability and the cortical representation of the paretic muscles occurs (Dimyan and Cohen, 2011). Consequently, TMS has been extensively explored to externally augment the excitability of the stroke-affected (ipsilateral) M1 through high-frequency stimulation to improve function. Numerous studies focused on ipsilateral M1 stimulation have documented at least one improvement in motor function after treatment (Dimyan and Cohen, 2011). In chronic phases, the unaffected (contralateral) hemisphere shows increased cortical excitability, an adaptive mechanism to compensate for functional deficits. An alternative strategy to improve motor function on the paretic side involves the downregulation of M1 excitability in the intact hemisphere. This approach would allow more normal functioning of the ipsilateral hemisphere (Figure 3B).

Adding to this, a comprehensive 2022 systematic review and meta-analysis by Gao et al. investigated the combination of intermittent theta-burst stimulation (a method of modifying cortical excitability with repetitive TMS stimulation) with exercise. They identified thirteen randomized controlled trials (RCTs) involving 334 patients. Primary endpoints were the Fugl-Meyer Assessment Scale (FMA), a stroke-specific, performance-based impairment index, and the Motor Assessment Scale (MAS), a clinical tool for measuring task performance related to daily living activities. The analysis found significant improvement in FMA scores when iTBS was combined with physical exercise. These scores reflect on coordinated and dissociative movements. However, they concluded that the positive effect of iTBS on motor function was only evident in chronic stroke patients, not in those in the subacute phase (Gao et al., 2022). These findings highlight TMS, particularly when paired with physical exercise, as a promising intervention for stroke rehabilitation. Its value lies in its potential as a standalone therapy and its complementary role when used alongside other treatments, such as physical therapy and occupational therapy, to optimize rehabilitation outcomes.

### Traumatic brain injury

Although there is limited research on Transcranial Magnetic Stimulation (TMS) following traumatic brain injury (TBI), preliminary pre-clinical and human pilot studies indicate that brain stimulation holds promise as a treatment for TBI-related motor deficits (Clayton et al., 2016). In the past, safety concerns regarding seizure susceptibility made the use of cortical stimulation in individuals with TBI somewhat controversial. However, it is now understood that the incidence of seizure relates more directly to the severity of injury rather than the stimulation itself (Dhaliwal et al., 2015), indicating that individuals with more severe brain injury are at an increased risk. In light of these findings, it is important to differentiate between mild and moderate/severe TBI in the approach to TMS. Those with mild TBI may respond differently and with decreased risk to these treatments than individuals with moderate to severe TBI; therefore, stratifying by TBI severity could enhance this technique’s therapeutic potential and safety profile.

Individuals sustaining mild TBI often report a constellation of physical, cognitive, and emotional/behavioral symptoms referred to as persistent post-concussion symptoms (Ryan and Warden, 2003). These symptoms can persist from months to years following injury (Bramlett and Dietrich, 2015), and include vertigo, dizziness, imbalance, and vision changes (Mucha et al., 2018). While the exact pathophysiology of persistent post-concussion symptoms are not entirely understood, increasing evidence suggests that disruptions in large-scale brain networks, particularly those associated with cognitive control, play a significant role (Sharp et al., 2014). Given the observed correlation between network dysfunction and mTBI symptoms (Churchill et al., 2019), neuromodulation of brain areas affected by mTBI is emerging as a promising approach for addressing persistent postconcussion symptoms (Buhagiar et al., 2020). Additionally, considering the broad cortical projections of the vestibular system as well as the interconnection between cognitive and affective networks, rTMS can be a powerful tool for influencing motion perception and postural control (Paxman et al., 2018). This opens an avenue for enhancing the treatment outcomes of balance and dizziness issues post-TBI, which are frequently managed by physical therapists. Furthermore, TMS has demonstrated promising results for postconcussive depression (Rao et al., 2019) and headache (Leung et al., 2018). The reduction of these symptoms could result in increased participation in traditional rehabilitation therapies, ultimately improving the overall effectiveness of therapy and patient outcomes.

Functional recovery following moderate to severe TBI requires interventions that vary depending on the time elapsed since the injury. In the acute phase, the focus of recovery should be neuroprotective, with the aim of minimizing neurologic damage. In contrast, during the chronic stages of TBI, the rehabilitation strategy shifts to suppressing maladaptive changes and fostering behavioral improvements (Demirtas-Tatlidede et al., 2012). TMS should be applied in a manner that complements these rehabilitation strategies, tailoring the approach to each stage of brain injury for optimal therapeutic benefit. Several experimental studies in animal models have explored the use of rTMS to enhance neuroprotection and neurorecovery after TBI with promising results (Lu et al., 2015, 2017; Verdugo-Diaz et al., 2017; Shin et al., 2018), however there are surprisingly few studies that have obtained substantial evidence regarding effects of rTMS in humans with TBI. Although rTMS has the potential to be a useful treatment for common TBI symptoms, brain injury is generally considered a contraindication to the repetitive forms of TMS due to the increased overall neural excitability and risk of seizures. This has led to TBI patients being excluded from most rTMS studies, making it challenging to accurately evaluate the safety and effectiveness of rTMS as a treatment for TBI (Rossi et al., 2009).

One standout clinical trial demonstrates the potential for rTMS to be combined with neurorehabilitation to enhance patient outcomes. This study explored the effects of rTMS paired with cognitive training on cognitive impairment in patients with traumatic brain injury (TBI) using multimodal magnetic resonance imaging (MRI) (Zhou et al., 2021). It included 166 patients with cognitive impairment post-TBI, dividing participants into two groups: one received rTMS combined with cognitive training (the observation group), while the other received cognitive training only (the control group). Results indicated that the observation group, who underwent rTMS, displayed significant improvements in various measures, such as the Glasgow Coma Scale (GCS) score, metabolic ratios examined by magnetic resonance spectroscopy imaging (MRSI), cognitive impairment score and grading, as well as the modified Barthel index. These results demonstrate the potential of rTMS as a viable adjunct to traditional rehabilitation therapy, amplifying the beneficial effects on cognitive impairment in patients post-TBI. However, while these findings are promising, particularly in cognitive rehabilitation, the use of rTMS in conjunction with motor rehabilitation in human subjects still requires further investigation.

### Spinal cord injury

When considering TMS for individuals with SCI, it is important to consider the differences between the use-case for complete vs. incomplete SCI. Patients with incomplete paraplegia generally have a good prognosis in regaining locomotor ability (∼76% of patients) within a year of injury (Waters et al., 1994), whereas individuals with complete spinal cord injury experience limited recovery of lower limb function, especially if their neurologic level of injury is above T9 (Waters et al., 1992). Therefore, TMS for functional motor recovery should be considered primarily for individuals with incomplete injury. On the other hand, TMS has also been investigated for its ability to reduce spasticity, a common disorder in patients with incomplete and complete SCI. Therefore, patients with complete SCI should not be excluded when considering future research clinical applications of TMS.

rTMS has demonstrated preliminary promise as an effective therapeutic modality for the recovery of motor function after spinal cord injury. In a 2004 study, Belci et al. examined TMS’s ability to promote somatomotor functional recovery in patients after incomplete SCI. The results indicated a short-term reduction in cortical inhibition with rTMS, and a temporary decrease in the activity of inhibitory neurons in the cerebral cortex. Reducing cortical inhibition can have various effects on brain function, including improving the function of certain brain circuits involved in movement. This study also reported a lasting improvement in sensory and motor function as per the ASIA scale, a tool used to assess the severity of SCI (Belci et al., 2004).

Additionally, a few studies have demonstrated the enhanced therapeutic potential of rTMS when combined with other therapeutic interventions. In a 2019 study, Krishan et al. aimed to test if rTMS promotes plasticity and rehabilitation in a rat model of acute vs. chronic SCI. The acute-TMS group demonstrated significant improvements in locomotor performance compared to chronic and no-TMS groups, indicating that rTMS therapy beginning in the acute phase after SCI promotes neuroplastic change. In a 2022 randomized control trial, Pulverenti et al. combined TMS with spinal cord stimulation during robotic-assisted locomotor training (Pulverenti et al., 2022). Their findings suggest pairing spinal cord stimulation with brain stimulation via TMS may further augment the benefits of locomotor training vs. locomotor training alone. Kumru et al. conducted two sham-controlled randomized controlled trials, combining high-frequency TMS with supervised gait training in groups of sub-acute SCI ambulators and marginal ambulators. The trials reported that rTMS has positive effects on the recovery of gait function and lower limb strength (Krogh et al., 2022). Overall, while these studies demonstrate the potential of rTMS in SCI rehabilitation, further research is needed before integrating this technique into clinical practice. Understanding the optimal dosage and timining of rTMS in combination with other physical therapy interventions will be critical to fully harnessing the benefits of this technology in the clinical setting.

## Discussion: TMS-enhanced rehabilitation

TMS shows significant promise as a neurostimulation technique to enhance neuroplasticity in various neurological conditions. By strategically integrating TMS with motor learning practices, their individual therapeutic effects could be amplified, as they demonstrate similar mechanisms for driving neuroplasticity. TMS can prime neuronal networks in the cortex when delivered prior to a task, while stimulation delivered simultaneously with the task can recruit specific sets of synapses involved in performance (Villamar et al., 2012). Based on the existing evidence, rTMS could potentially play a role in improving the effectiveness of other rehabilitation treatments (Formica et al., 2021). However, to effectively utilize TMS as a complementary therapy, the specific neurological characteristics of each condition and diagnosis must be considered. For instance, high-amplitude training programs such as LSVT BIG and PWR inherently demand large movement patterns, making it challenging to conduct TMS therapy alongside this exercise intervention. In this case, it may be more beneficial to prime the nervous system with TMS prior to administering the exercise intervention.

Conversely, upper extremity CIMT for stroke rehabilitation, which can be completed in a seated position, may be more appropriately combined simultaneously with TMS. Importantly, TMS studies need to provide more precise information about stimulation location. For instance, while TMS over the motor cortex (M1) has shown promising effects on motor learning, stimulation of other regions, such as the cerebellum, might yield different results. See Table 1 for a summary of potential clinical applications and future areas for research. Further research is needed to derive ideal parameters, timing, and application of TMS to prescribe personalized therapeutic interventions.

**TABLE 1:** Potential clinical applications for TMS + neurorehabilitation.

Diagnosis	Potential clinical application for TMS therapy	Potential combination of TMS with rehabilitation	Clinical readiness
Parkinson’s disease	High frequency, bilateral, multi-session rTMS over M1 (Yang et al., 2018)	Priming with TMS prior to large-amplitude training (Fox et al., 2012; Osborne et al., 2022)	Needs more research
Stroke	iTBS for individuals with chronic stroke (Gao et al., 2022)	Concurrent iTBS with UE CIMT therapy (Nesin et al., 2019)	Needs more research
TBI	rTMS for management of mild TBI symptoms (Buhagiar et al., 2020)	Priming with TMS prior to vestibular therapy (Schlemmer and Nicholson, 2022)	Needs more research
SCI	rTMS combined with neuromuscular stimulation for individuals with incomplete SCI (Belci et al., 2004)	Concurrent rTMS with NMES during LE biking (FES bike) (van der Scheer et al., 2021)	Needs more research

## Peripheral nerve stimulation (PNS)

### Overview

**FIGURE 4:**
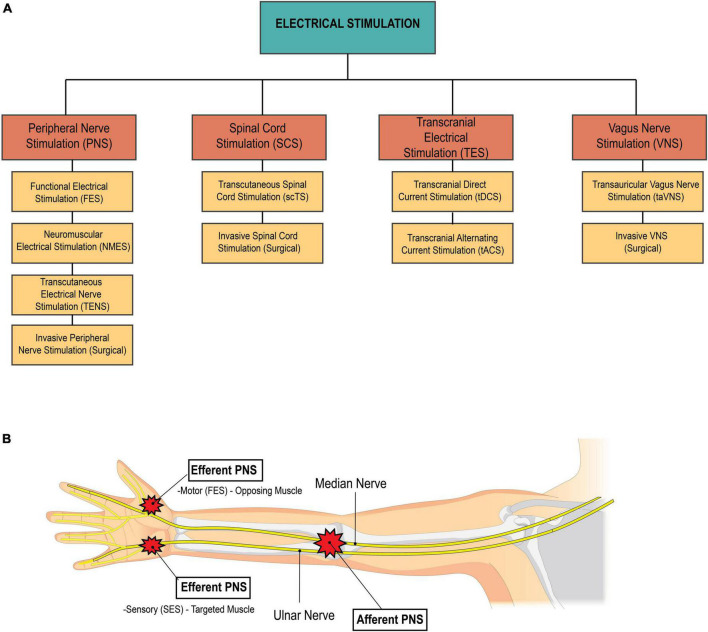
**(A)** Breakdown of types of electrical stimulation. The four main categories of electrical stimulation used in medical management of disease and injury include peripheral nerve stimulation, spinal cord stimulation, transcranial electrical stimulation, and vagus nerve stimulation. **(B)** Afferent vs. efferent PNS. Afferent PNS involves stimulation proximally to effect distal structures. For example, stimulation of the median and radial nerves travels down the arm to effect hand function. On the other hand, efferent PNS involves stimulation at or near the target structure. The two types of efferent PNS are sensory electrical stimulation and motor electrical stimulation. Sensory electrical stimulation (SES) is applied on the targeted muscle at intensities below the motor threshold. functional electrical stimulation (FES), a type of motor stimulation, is applied to the muscle opposing the desired action, at intensities at or above the motor threshold. Source: Biorender.com.

Peripheral nerve stimulation (PNS) is a type of non-invasive neuromodulation that delivers electrical stimuli to targeted peripheral nerves. PNS is traditionally used in physical therapy clinics for pain management, however with varied parameters and settings, PNS serves as a potential therapeutic modality to aid in the rehabilitation of neurological conditions. PNS techniques such as neuromuscular electrical stimulation (NMES) and functional electrical stimulation (FES) facilitate voluntary muscle contractions, and can be strategically used in the rehabilitation setting for muscle re-education (Price and Pandyan, 2000; Ada and Foongchomcheay, 2002; Ragnarsson, 2008; Sujith, 2008). NMES & FES specifically target lower motor neurons (LMNs) to produce a muscle contraction. NMES involves the application of electrical stimuli to a muscle, with the goal of improving muscle strength, reducing muscle atrophy, and facilitating motor recovery (Chipchase et al., 2011). The stimulation is used to artificially generate a muscle contraction in the absence of voluntary muscle control or to augment a weak voluntary muscle contraction. On the other hand, FES is typically used to enable functional movements or tasks. The electrical stimulation in FES is often synchronized with a specific task or movement to assist with function. Refer to Figure 4A for an overview of different types of electrical stimulation used in the clinical setting.

Importantly, these PNS techniques also promote neuroplasticity by increasing the basal level of spinal excitability, leading to improved voluntary motor function even with lower input levels (Grahn et al., 2017). Additionally, emerging research suggests that electrical stimulation techniques like NMES and FES can enhance cortical plasticity by increasing the corresponding motor and sensory cortical representation areas (Chipchase et al., 2011). Therefore, PNS can be integrated with traditional rehabilitation methods to amplify neuroplastic changes and enhance outcomes in patients with neurological conditions. Further research to explore these potentials can open new avenues for personalized interventions, enhancing patient outcomes in various neurological conditions.

## PNS implication by disease

### Parkinson’s disease

PD patients can exhibit a variety of motor symptoms, with resting tremors being one of the most significant. Studies have shown that 69% of patients have resting tremor at the onset of disease, and 75% develop tremor during the course of the disease (Louis and Machado, 2015). Hand tremors can significantly affect quality of life (Meng et al., 2022), and are commonly treated with oral medications or invasive surgery. However, non-invasive electrical stimulation methods have emerged as effective alternative for tremor reduction (Meng et al., 2022).

PNS techniques for tremor reduction falls into one of two categories: *efferent* PNS or *afferent* PNS. *Efferent* PNS involves stimulating the targeted muscle or its antagonist to reduce tremor (Figure 4B). Sensory electrical stimulation (SES), a subtype of efferent PNS, involves stimulation to the targeted muscle below motor threshold. This acts to suppress or regulate the neuronal pathway involved in tremor (Raethjen et al., 2000; Xu et al., 2016; Dideriksen et al., 2017). Conversely, functional electrical stimulation (FES), which is another form of efferent PNS, involves stimulation above the motor threshold to the antagonist, which leads to active muscle contraction to control tremor. *Afferent* PNS involves stimulation of the afferent nerve fibers of the radial and median nerves to inhibit muscles related to tremor (Figure 4B; Meng et al., 2022). All three of these PNS strategies have shown promise in reducing tremor and are comparable or superior to first-line pharmacotherapies. FES appears to be the most effective of these strategies (Meng et al., 2022), yielding an average rate of tremor inhibition of over 50% (Popović Maneski et al., 2011; Zhang et al., 2011; Gallego et al., 2013; Freeman et al., 2015). However, SES may be a more comfortable alternative for tremor suppression. In clinical settings, these PNS techniques could potentially be used to reduce tremor before or during exercise or functional training sessions, improving motor control and accuracy of movements and enhancing physical therapy interventions.

### Stroke

PNS can be paired with functional training in individuals with stroke, enhancing neuroplasticity and improving motor function outcomes, highlighting its applicability in a rehabilitation setting (Kim et al., 2020). For example, in 2012, Ikuno et al. investigated PNS combined with task-oriented training in 22 stroke patients by combining PNS of median and ulnar nerves with task-oriented training for one week. Combined PNS and task training significantly improved hand function as compared to the group with task-only training (Ikuno et al., 2012). Fleming et al. (2015) found that an experimental group that underwent both task-oriented training and peripheral nerve stimulation (PNS) to median, ulnar, and radial nerves showed a positive effect on upper extremity (UE) function compared to a control group that received task-oriented training alone (Fleming et al., 2015). In a 2020 study, Kim et al.’s included 29 patients with hemiplegia, with 14 subjects receiving PNS + task-oriented training for 4 weeks, while 15 control group subjects underwent only task-oriented training. After 4 weeks, muscle activity of extensor carpi radialis, flexor carpi radialis, grip strength, and Action Research Arm Test were significantly higher in the experimental group (Kim et al., 2020). Collectively, these studies validate the approach of combined electrical stimulation of peripheral nerves timed with functional movements after stroke to enhance outcomes. However, an additional review by Quandt and Hummel (2014), proposes that while there are studies indicating the potential benefit of FES in stroke rehabilitation, more homonogenous randomized controlled trials are needed to generate stronger evidence supporting an advantage of FES over traditional rehabilitation approaches (Quandt and Hummel, 2014).

However, the application of PNS improving lower extremity function may be more challenging. This is reflected in a 2021 systematic review, where Cunha et al. evaluated the effectiveness of FES applied to the paretic peroneal nerve in individuals with foot drop secondary to stroke, including fourteen studies with data from 1115 participants. Their analysis revealed low-quality evidence of positive effects of FES on gait speed when combined with physical therapy (Jaqueline da Cunha et al., 2021). Therefore, further investigation and standardization of PNS protocols combined with gait training is warranted.

### Traumatic brain injury

Following a traumatic brain injury, the excitability of the motor cortex near the injury site may be substantially decreased, causing a reduction in cortical map representations of the muscles that are affected (Traversa et al., 1997; Bütefisch et al., 2006). While FES and NMES have been studied extensively in stroke, less work has been done for individuals with TBI. In a 2021 case report, Milosevic et al. delivered functional electrical stimulation therapy (FEST) combined with task-specific and repeated training to an individual with chronic TBI with the assistance of a therapist. The results suggested that task-specific and repetitive FES can effectively increase cortical recruitment (Milosevic et al., 2021). In a 2010 study, 30 participants in an inpatient setting with either stroke or TBI resulting in hemiparesis received either real FES cycling or sham-FES cycling as a treatment intervention. Electrodes were applied to bilateral quadriceps, hamstrings, glutes, and tibialis anterior, 5x/week for 4 weeks. Results demonstrated improved LE strength and gait speed after FES treatment (Ambrosini et al., 2011). However, this clinical trial did not differentiate between stroke and TBI diagnosis, and results should be considered cautiously. Although FES and TENS are recommended as adjunct treatments in published clinical practice guidelines (CPGs) for treatment of TBI, further studies are warranted to determine the efficacy of PNS as a treatment (Lee et al., 2019).

PNS has also been explored as a potential treatment for muscle wasting in critically ill TBI patients during their ICU stay. In this context, NMES has been identified as a promising approach to alleviate the functional and clinical effects of muscle wasting. Silva et al. (2019) conducted a study to evaluate the effectiveness and duration of an NMES protocol on muscle architecture, neuromuscular electrophysiological disorder (NED), and muscle strength. Their findings indicate that NMES administered for fourteen consecutive days was effective in reducing muscle atrophy, decreasing the incidence of NED, and mitigating muscle weakness in critically ill TBI patients (Silva et al., 2019). While this study did not pair NMES with traditional physical or occupational therapy, it hints at the potential benefits of a more integrated approach. Specifically, in patients with acute and severe TBI, traditional therapy could be enhanced by pairing NMES with functional, low level tasks such as bed mobility and transfer training. However, integrating PNS with traditional therapeutic approaches during the acute phase of severe TBI presents several challenges. The medical instability of patients immediately after traumatic incident often precludes additional interventions such as PNS. Additionally, cognitive deficits and communication difficulties characteristic of severe TBI may hinder patients’ understanding of or cooperation with the therapy. Future research could illuminate the possible synergies between NMES and task-oriented therapy, and address the barriers of implementing this approach in this population.

### Spinal cord injury

SCI causes damage to both the pathways of efferent and afferent neurons, which include the descending motor fibers from the brain to the spinal motor neurons and the ascending somatosensory fibers from the PNS through the spinal cord and back to the brain (Hamid and Hayek, 2008). Electrical stimulation in individuals with SCI is believed to work by inducing neuroplastic changes at synapses within the spinal cord (Karamian et al., 2022), and it plays a prominent and important role in rehabilitation following SCI. Studies have demonstrated that FES can enhance muscle power output and resistance (Gorgey et al., 2018), with various research supporting its effectiveness in the recovery of upper extremity function after SCI (Popovic et al., 2011). FES shows promise for both acute and chronic SCI, as indicated by an RCT in individuals with cervical incomplete SCI (Kapadia et al., 2011). FES was applied 5 days per week for 8 weeks and compared to conventional occupational therapy targeting improvement of voluntary upper limb function. Participants receiving FES therapy showed greater improvements in hand function at discharge, as well as 6-month follow-up, compared to the control group (Kapadia et al., 2011). FES cycling can improve lower extremity function as well, indicated by a 2022 review where Scheer et al. identified ninety-two FES cycling exercise intervention studies, including 999 individuals with SCI. They concluded that FES cycling substantially improved lower body muscle health in adults with SCI (van der Scheer et al., 2021).

The flexible nature of FES protocols for SCI can be adopted to either in-patient our outpatient settings, expanding its reach and application. However, barriers do exist to implementing electrical stimulation in individuals with SCI, and should be considered. Autonomic dysreflexia (AD), a potentially lifethreatening condition characterized by an exaggerated response of the autonomic nervous system that results in a sudden and significant increase in blood pressure, can be instigated by various stimuli below the level of injury. For example, AD can be triggered by bowel or bladder distension, pressure sores, or other forms of irritation such as the application of electrical stimulation. Therefore, close monitoring of the patient’s cardiovascular response during therapy is crucial to ensuring patient safety by detecting and managing any instances of AD. Despite these concerns, the potential benefits of electrical stimulation in individuals with SCI propose an avenue for further exploration and clinical application.

Additionally, transcutaneous spinal cord stimulation (tSCS) has emerged as another approach to neuromodulation to treat patients with SCI, which is distinct from peripheral nerve stimulation (PNS), but shares its non-invasive delivery method and focuses on a specific anatomical target. The technique involves the application of electrical stimulation to the skin overlying the spinal cord, allowing for indirect modulation of spinal cord activity (Field-Fote, 2004). In the case of SCI, tSCS may provide more significant functional recovery than rehabilitation alone (Tefertiller et al., 2022; Tharu et al., 2023), especially in cases of complete SCI, where the signal is obstructed from descending below the level of the injury. While some circuits are spared in patients with complete SCI, these circuits are frequently insufficient to create a satisfactory level of excitability for stimulating motor neurons below the level of injury. Electrical stimulation at the level of the spinal cord could help strengthen spared neural circuitry in facilitation of adequate stimulation of motor neurons for muscle contraction (Harkema et al., 2011). Pairing this innovative approach with traditional rehabilitation techniques could provide enhanced outcomes for patients with SCI. The continuous exploration and refinement of these methodologies are important in realizing the full potential of SCI recovery.

## Discussion: PNS-enhanced rehabilitation

In summary, PNS can be an effective adjunct intervention to enhance neuroplasticity in individuals with neurologic conditions. Unlike many other neurostimulation devices, handheld and portable peripheral nerve stimulation devices are readily available in physical therapy clinics, offering an accessible and safe tool for improving patient care (Kaye et al., 2021). While most therapists are familiar with using passive TENS for pain relief after orthopedic injuries, an expanded understanding of various applications of PNS is warranted. Clinicians should consider disease-specific implications, types of electrical stimulation, timing of stimulation, and stimulation parameters to utilize PNS to enhance neuroplasticity and improve motor outcomes in individuals with neurologic diagnoses. See Table 2 for a summary of potential clinical applications and future areas for research. Despite the substantial body of evidence supporting the use of PNS in rehabilitation, further examination of PNS and its role in neurologic recovery is needed, with specific focus on the timing, underlying mechanisms, and neural targets to enhance patient outcomes. By integrating PNS with traditional rehabilitation methods, we can capitalize on new avenues for enhancing patient recovery and outcomes in various neurological diagnoses.

**TABLE 2:** Potential clinical applications for PNS + neurorehabilitation.

Diagnosis	Potential clinical application for PNS therapy	Physical exercise recommendations	Clinical readiness
Parkinson’s disease	FES for tremor reduction (Meng et al., 2022)	FES combined with UE functional training (Popa and Taylor, 2015)	Needs more research
Stroke	Afferent PNS to median, radial, and ulnar nerves of paretic UE (Fleming et al., 2015)	PNS combined with repetitive task practice (Fleming et al., 2015; Kim et al., 2020)	Ready for implementation, more research needed for optimization
TBI	Chronic TBI–FES for patients with hemiplegia (Ambrosini et al., 2011; Milosevic et al., 2021) Acute TBI–NMES for critically ill patients (Silva et al., 2019)	Chronic TBI–FES combined with repetitive task practice (Lee et al., 2019) Acute TBI – NMES combined with functional sit < > stand training (Canning et al., 2003)	Ready for implementation, more research needed for optimization
SCI	FES for acute, chronic, incomplete and complete SCI (Mangold et al., 2005; Kapadia et al., 2011; van der Scheer et al., 2021)	FES combined with UE and/or LE cycling (van der Scheer et al., 2021)	Ready for implementation

## Vagus nerve stimulation (VNS)

### Overview

**FIGURE 5:**
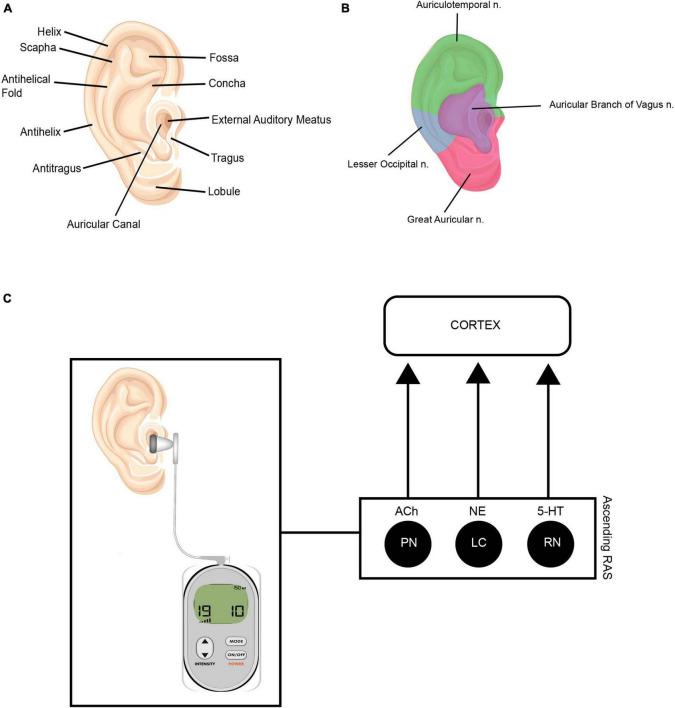
**(A)** Anatomy of the outer ear. **(B)** Innervation of outer ear. Illustrations depicting the auricular branch of the vagus nerve (ABVN—represented in purple) innervates the external meatus, which is the area targeted during taVNS treatment. **(C)** This modulates the ascending reticular activating system (RAS). The ascending RAS includes the pedunculopontine nucleus (PPN), the locus coeruleus (LC) and raphe nuclei (RN), and modulates acetylcholine (Ach), norepinephrine (NE) and serotonin (5-HT) to higher-order brain structures to modulate attention and regulate awareness, arousal and sleep. Source: Adobe Stock.

Vagus Nerve Stimulation (VNS) is a neuromodulation technique that involves applying electrical stimulation to the vagus nerve, or 10th cranial nerve. Traditional VNS is delivered via a surgically implanted device, and has now been FDA approved for clinician use in treatment of migraine, cluster headache, and depression. VNS is thought to affect various brain regions through direct effect on nucleus tractus solitarius (NTS) and locus coeruleus (LC). This activation leads to rapid activation of cholinergic and noradrenergic systems, resulting in enhanced neuroplasticity associated with coincident events (Engineer et al., 2019). VNS also increases levels of brain-derived neurotropic factor (BDNF) which is a key regulator of neuroplasticity (Follesa et al., 2007). Given the anatomical distribution of the vagus nerve and its close location to the skin surface, non-invasive methods have recently been developed, with delivery sites at the neck or via stimulation of superficial projections of the vagus nerve in the outer ear. Transauricular Vagus Nerve Stimulation (taVNS) involves stimulation of the auricular branch of the vagus nerve (Wang et al., 2021) via the cymba conche (see Figure 5).

Compared to traditional VNS, non-invasive VNS is preferred to minimize the risk of infection, postoperative complications, device rejection, and patient discomfort. Additionally, taVNS is inexpensive, portable, and safe, making it an appealing device for rapid translation of VNS research into clinical practice. Considering the wide range of effects that VNS has on neural circuits, this non-invasive method is an excellent adjunct to traditional rehabilitation. By modulating the patient’s neural pathways, VNS could augment the efficacy of conventional physical and occupational therapy protocols, offering improved therapeutic outcomes.

Engineer et al. conducted an initial study to determine the effectiveness of VNS in enhancing cortical plasticity (Engineer et al., 2011). They aimed to investigate whether the presentation of tones paired with VNS could induce reorganization of the tonotopic map in the primary auditory cortex of mice. The results indicated that repeated presentation of tones without VNS did not lead to map reorganization. However, presenting the same number of tones coupled with VNS considerably increased the number of neurons that reacted to corresponding tone frequencies. This suggests that combining brief bursts of VNS with sensory or motor events can lead to potent and event-specific plasticity in neural circuits (Hays, 2016), and has formed the theoretical background for integrating VNS into personalized therapy regimens. These findings highlight the potential for VNS, when combined with specific sensory and motor stimuli during rehabilitation, to drive targeted neuroplastic changes and enhance therapeutic outcomes. As the field of personalized medicine continues to evolve, more research is needed to determine the precise timing and application of VNS to optimize the benefits of this approach.

## VNS implication by disease

### Parkinson’s disease

VNS has been investigated for use in the treatment of motor and non-motor symptoms of Parkinson’s Disease. While PD has traditionally been associated with the loss of dopaminergic cells in the substantia nigra, recent studies have suggested that the Locus Coeruleus Norepinephrine (LC-NE) system may also play a significant role in the pathophysiology of this condition (Rommelfanger and Weinshenker, 2007). The locus coeruleus (LC) is a brainstem structure that produces much of the brain’s norepinephrine (NE), and degradation of the neurons of the LC occurs with Parkinson’s disease (German et al., 1992; Hoogendijk et al., 1995; Marien et al., 2004). VNS stimulates the neurons of the LC, suggesting that stimulating LC output with VNS has the potential to counteract effects of LC degradation in PD (Farrand et al., 2020).

In a 2020 animal study, Farrand et al. determined that VNS results in reduced inflammation, attenuated LC and SN phenotypic neuronal loss, and enhanced motor function (Farrand et al., 2020), suggesting that stimulating LC output with VNS has the potential to modify disease progression of PD. Jiang et al. also evaluated the effects of VNS on SN-DA neurodegeneration and associated neuroinflammation & immune responses in a rat PD model, concluding that taVNS exerts neuroprotective effects against dopaminergic damage possibly by suppressing evolution of inflammation (Jiang et al., 2018). Kin et al. confirmed this again in a 2021 study, demonstrating that VNS w/0.25–0.5 mA intensity preserves dopamine neurons, reduces inflammatory glial cells, and increases noradrenergic neurons (Kin et al., 2021).

More recently, scientists have begun investigating using taVNS in a clinical setting to enhance PD rehabilitation. In 2022, Marano et al. published results from a pilot-controlled study with a double-blind randomized crossover design, where they investigated the effects of taVNS on the gait of 12 patients with idiopathic Parkinson’s Disease. Participants were randomly assigned into two groups: active taVNS and control. The results showed statistically significant changes in stride length, swing amplitude, gait speed, and gait time after taVNS (Marano et al., 2022). Further studies are warranted to determine efficacy of combining taVNS with rehabilitation to improve motor and non-motor symptoms of Parkinson’s Disease in humans.

### Stroke

After stroke, the brain begins to reorganize, undergoing adaptive neuroplastic changes to compensate for the loss of previously functional neural pathways. The goal of motor rehabilitation after stroke is to facilitate positive adaptive functional changes, reinforcing the connection between the impaired brain region and corresponding extremity. Additionally, rehabilitation aims to prevent maladaptive neuroplasticity, which can lead to overreliance on the unaffected limb and learned non-use of the affected limb. Given VNS’s ability to enhance neuroplasticity, VNS has been investigated as a therapeutic approach to enhance positive neuroplasticity during stroke recovery. Khodaparest et al. explored this theory by utilizing a rat model of ischemic stroke (Khodaparast et al., 2013). The rats were initially trained on the bradykinesia assessment task, which is a quantitative measurement of forelimb movement speed, before receiving a cortical ischemic lesion (Hays et al., 2013). They were then divided into two groups for rehabilitative training, with one group receiving VNS on successful trials over 5 weeks and the other group without VNS. They found that VNS combined with rehabilitative training fully restored forelimb performance by the second week of training and significantly enhanced recovery compared to rehab training without VNS. In a follow up study, this same group found that VNS paired with rehabilitative training resulted in greater recovery of forelimb strength as compared to training without VNS. These results also suggested long-term improvement in forelimb strength, indicating persisting results after cessation of VNS therapy (Hays et al., 2014b). Hays et al. also investigated VNS paired with rehabilitative training after hemorrhagic stroke (Hays et al., 2014a), as plasticity within spared circuitry is believed to support recovery after intracerebral hemorrhage (ICH) (Auriat et al., 2010; Santos et al., 2013). They found that VNS paired with rehabilitative training resulted in significantly improved forelimb function when compared to pre lesion levels, however unlike the complete recovery observed in ischemic stroke, VNS therapy failed to fully restore forelimb function.

Based on these preliminary animal studies, Dawson et al. evaluated VNS paired with physical rehabilitation in a pilot study in stroke patients (Dawson et al., 2016). Over 6 weeks, they combined VNS with standard rehabilitative tasks. Patients who underwent this combination showed a notable improvement in the change of UE Fugl-Meyer score as compared to patients who received the same rehabilitation but without VNS. Furthermore, in 2021, this same group conducted a pivotal multi-site randomized controlled trial (VNS-REHAB), where they paired rehabilitation with active or sham vagus nerve stimulation in individuals with moderate to severe arm weakness (Dawson et al., 2021). The individuals receiving active VNS demonstrated a mean FMA-UE score improvement of 5 points, compared to 2 points in the control group. This study demonstrated that participants with moderate to severe arm impairment after ischemic stroke showed clinically meaningful improvements in motor impairment and function with paired VNS compared to rehabilitation alone. In a 2017 study, Capone et al. (2017) explored whether non-invasive VNS combined with VNS can enhance upper limb functionality in chronic ischemic and hemorrhagic stroke by delivering taVNS for 10 working days. They found that the treatment was safe and tolerable, and FMA scores were significantly better in the real group compared to sham. Wu et al. (2020) evaluated 21 subacute ischemic stroke patients, who were assigned to rehab + taVNS or rehab + sham for 15 consecutive days, finding that scores were significantly higher than before treatment in all groups, and there was a significantly greater improvement of those measures in the taVNS group compared to the sham taVNS group. More recently, Li et al. (2022) showed that taVNS combined with conventional rehab was effective in treating stroke for up to 1 year after intervention, suggesting long term retention of benefits. In a 2021 review, Morris et al. found that VNS was most effective when paired coincident with or immediately after movements during rehabilitation, with the most ideal stimulation parameters being 0.8 mA, 30 Hz, and 100 μs, providing guidelines for future taVNS + rehabilitation studies.

These studies provide the basis for a compelling argument to incorporate VNS into clinical practice to enhance standard rehabilitation. However, several barriers need to be addressed. For example, optimal stimulation parameters have not yet been determined, and there is a paucity of evidence comparing various stimulation parameters when combined with rehabilitation. Further exploration and refinement of VNS parameters are needed to maximize functional outcomes. Additionally, patient-specific variables including the nature and severity of stroke, individual neuroplastic potential, and comorbid conditions can influence the efficacy of VNS. This highlights the need for a personalized approach in the application of VNS in stroke rehabilitation.

### Traumatic brain injury

Recovery of motor function after moderate to severe TBI, similar to stroke, is linked to plasticity in surviving motor circuits (Nishibe et al., 2010). Therefore, VNS therapy combined with rehabilitation has the potential to improve plasticity and aid in recovery. To investigate this hypothesis, Pruitt et al. evaluated whether VNS therapy could improve recovery of motor function in a controlled cortical impact model of severe TBI (Pruitt et al., 2016). VNS was paired with rehabilitative therapy over a period of 5 weeks, with results showing VNS paired with rehabilitative training significantly improved recovery of volitional forelimb strength compared with rehabilitative training without VNS after TBI. In addition to enhancing plasticity, Tang et al. concluded that VNS significantly ameliorated tissue damage, neurological deficits, and cerebral edema compared with a sham VNS group in animal TBI models (Tang et al., 2020).

Human studies are sparse, as patients with TBI are commonly excluded from VNS studies due to seizure risk. However, in a 2012 case control study, Englemont et al. retrospectively compared seizure outcomes after VNS therapy in patients with post-traumatic epilepsy (PTE) vs. those with no PTE. They found that after VNS therapy, patients with PTE demonstrated greater reduction in seizure frequency than patients without PTE, concluding that VNS can help reduce seizure frequency (Englot et al., 2012). Hakon et al. evaluated the feasibility of taVNS in 5 patients presenting with diffuse axonal injury one month after severe TBI, demonstrating that taVNS is a feasible and safe VNS strategy for patients following severe TBI (Hakon et al., 2020). Similarly, Noe et al. enrolled chronic adult patients with disorders of consciousness (DOC) after severe TBI, providing forty 30 min transauricular VNS sessions 2x/day, 5x/week. They concluded that taVNS could be a safe and effective tool to facilitate consciousness recovery in severely brain-injured patents (Noé et al., 2020).

These findings indicate that VNS, particularly taVNS, can be a safe and potentially effective strategy for patients following TBI. However, the body of evidence supporting the integration of this intervention into clinical practice is sparse and preliminary. More robust studies are needed to determine the most optimal way to integrate VNS with rehabilitation interventions for individuals with TBI. Potential barriers to integration include the complexity of TBI pathology and individual variability among patient presentation, making it challenging to establish a universal treatment protocol. Further research is essential to navigate barriers and identify the best strategies to combine VNS with rehabilitation for individuals with TBI.

### Spinal cord injury

As previously discussed, damaged synaptic connectivity results in impaired motor function after SCI, particularly in cases of motor complete injury (Varma et al., 2013). This means that there is a disruption of neural pathways responsible for transmitting motor commands from the brain to the relevant muscles. Therefore, therapies such as VNS that enhance plasticity could potentially improve functional outcomes after SCI by reinforcing these neural pathways. Ganzer et al. (2018b) investigated this hypothesis by training rats on a reach-and-grasp task and then inducing SCI. The rats were divided into three groups who all went through physical rehabilitation. Two of the three groups also received VNS, with one group receiving stimulation after their best trials and one group receiving stimulation after their worst trials. This type of activity-dependent stimulation is called closed-loop vagus stimulation (CLV). Their findings showed that an improvement in recovery was noticeable only when the stimulation was combined with best-effort trials that were close to the intended outcome. This highlights the importance of appropriately timing the stimulation to shape the behavioral outcomes and optimize recovery. Darrow et al. also investigated closed loop VNS combined with rehabilitation training after SCI, demonstrating VNS + rehab training significantly improved recovery of volitional forelimb strength compared to equivalent rehabilitative training without VNS (Darrow et al., 2020). Surprisingly, VNS dependent enhancement of recovery was also able to be generalized to two similar (untrained) forearm tasks, which has significant implications for future clinical trials.

These findings highlight the potential of VNS as a powerful adjunctive treatment for SCI rehabilitation. They attest to the effectiveness of VNS for enhancing functional recovery, but also emphasize the need for precise timing and strategic application of this stimulation. However, further investigations are needed to refine these approaches.

## Discussion: VNS enhanced rehabilitation

In summary, mounting evidence from animal models and pilot clinical trials indicates that VNS paired with rehabilitative training can enhance the benefits of neuroplastic rehabilitative interventions. More specifically, succinctly pairing VNS with successful motor trials can optimally enhance plasticity, positively shaping behavior and improving rehabilitative outcomes. See Table 3 for a summary of potential clinical applications and future areas for research.

**TABLE 3:** Potential clinical applications for VNS + neurorehabilitation.

Diagnosis	Potential clinical applications for VNS therapy	Physical exercise recommendations	Clinical readiness
Parkinson’s disease	VNS during high amplitude training (Marano et al., 2022)	LSVT BIG therapy*, PWR Moves (Fox et al., 2012; Osborne et al., 2022) *VNS applied during most successful trials of Maximal Daily Exercises	Needs more research
Stroke	VNS during repeated task practice (Hays et al., 2014a,b; Dawson et al., 2016, 2021)	Constraint Induced Movement Therapy*(Nesin et al., 2019) *VNS applied during shaping tasks, most successful trials	Needs more research
TBI	VNS combined with rehabilitation (Pruitt et al., 2016; Hakon et al., 2020; Noé et al., 2020)	Mild TBI–VNS to decrease symptom provocation before, during, or after vestibular rehabilitation (Schlemmer and Nicholson, 2022) Severe TBI–VNS during sensory stimulation for coma recovery (Thibaut et al., 2019); stimulate when patient attends to stimulus	Needs more research
SCI	VNS during functional task training, gait training, or cycling (Ganzer et al., 2018b; Darrow et al., 2020)	FES combined with UE and/or LE cycling*(van der Scheer et al., 2021)*VNS applied during peak power output	Needs more research

A significant barrier to the incorporation of non-invasive VNS into clinical practice lies in the engineering challenge of synchronizing stimulation with specific patient movements. Current devices typically utilize an ear-clip, with variable stimulation sites. They lack a precise trigger mechanism for activating stimulation at the point of successful patient movement, which as discussed is a critical parameter for optimal neuroplastic enhancement. As such, we advocate for advancements in the engineering of taVNS devices, prioritizing the creation of responsive triggers that could be activated by therapists or potentially automated through the detection of successful movement parameters.

Additionally, there is a pressing need to optimize the parameters for non-invasive VNS, as current guidelines largely extrapolate from invasive VNS studies. However, it is critical to acknowledge that non-invasive and invasive VNS parameters may significantly differ due to variances in delivery methods, and should be explored independently. A comparative study between non-invasive and invasive VNS procedures would also provide valuable insights, facilitating an informed choice of technique based on efficacy, safety, patient comfort, and feasibility. An in-depth understanding of the underlying mechanisms of VNS will further highlight its clinical utility. While non-invasive VNS, particularly taVNS, holds significant potential for wider acceptance and translation into clinical practice, it is evident that substantial work lies ahead. From engineering advancements in devices to refined parameter settings and comprehensive studies into mechanisms of action, these steps are crucial to fully unlock the promise of VNS in neurorehabilitation.

## Future directions in neuromodulation

In addition to the neuromodulatory approaches explored in this review, it is also worth noting other emerging technologies that show promise in rehabilitation. Transcranial Direct Current Stimulation (tDCS) and Focused Ultrasound (FUS) are among such techniques that warrant further exploration.

### Transcranial direct current stimulation

Transcranial Direct Current Stimulation (tDCS) is a non-invasive brain stimulation technique that involves the delivery of a low-amplitude current between electrodes placed on the scalp. The current generates a weak electric field across the cortex, modulating neuronal activity by altering the resting membrane potentials (Datta et al., 2009; Salvador et al., 2010). This technique is portable, cost-effective, and relatively easy to administer, making it an attractive option for clinicians. tDCS has been shown to influence various cognitive processes and motor functions (Coffman et al., 2012; Parazzini et al., 2014), potentially offering a complementary approach in rehabilitation therapies.

Evidence demonstrating the clinical applicability of tDCS predominantly comes from stroke studies. In a systematic review, Marquez et al. analyzed 15 studies on the use of tDCS for stroke recovery. The methodological quality of the included studies was consistently high, and most studies reported positive effects of tDCS on motor function and impairment immediately after the intervention. However, there were limited long-term follow-up data available. They concluded that while tDCS shows promise as a therapeutic treatment for improving motor function in adults with residual motor impairments due to stroke, further research is needed to determine its long-term effectiveness (Marquez et al., 2015). Additionally, Kang et al. performed a systematic review and meta-analysis that found that tDCS can significantly improve motor learning post-stroke, measured by various motor function tests (Kang et al., 2016). However, they also acknowledge that further research is needed to determine optimal stimulation protocols and long-term effects.

The effectiveness of tDCS in enhancing motor function in Parkinson’s Disease is a matter of ongoing debate, with one review indicating beneficial outcomes (Pol et al., 2021), and another asserting that the available evidence remains inconclusive (Liu et al., 2021). Both, however, articulate the importance of further research to determine optimal tDCS parameters for functional recovery. Additionally, Kim et al. suggest that tDCS may be beneficial to patients with TBI for neuroprotection or functional recovery. However, they state that the implementation of more robust clinical trials are needed to confirm the efficacy of tDCS in this patient population and determine the most effective stimulation patterns (Kim et al., 2019). Overall, the precise mechanisms of action of tDCS are complex and multifaceted, warranting a more thorough understanding for its safe and effective implementation in clinical settings.

### Focused ultrasound

FUS is another non-invasive brain stimulation method that employs the propagation of acoustic waves to modulate neuronal activity (Kim et al., 2014; Tyler et al., 2018). It is similar to traditional medical imaging and diagnostic ultrasound techniques, however it utilizes acoustic waves that are concentrated to a specific target within the brain. Because of this similarity, FUS holds the potential for easy integration into existing physical therapy methods. Low-intensity pulsed ultrasound stimulation (LIPUS) has demonstrated a neuroprotective effect after brain injury (Bretsztajn and Gedroyc, 2018). Additionally, it has been observed to increase BDNF and VEGF expression in astrocytes while inhibiting cell apoptosis (Yang et al., 2015; Su et al., 2017). This evidence points to the potential use of FUS as a novel approach for clinical stroke treatment. Preliminary preclinical evidence indicates that FUS is safe and has beneficial neuromodulatory effects on motor behavior in Parkinson’s Disease (Lee et al., 2022). However, given the safety concerns associated with the use of focused ultrasound in humans, such as potential tissue heating and damage (Pérez-Neri et al., 2022), it requires rigorous research to establish its efficacy and safety profiles.

While these technologies were outside the scope of the present review, they nonetheless present promising possibilities for the future of physical therapy. As our understanding of these techniques continues to grow, and as further research elucidates their potential benefits and risks, it is likely that they will play a more central role in the repertoire of therapeutic neuromodulation approaches available to clinicians and researchers.

## Summary

In light of the evidence presented, exercise and physical activity clearly influence neuroplastic changes in individuals with various neurologic diagnoses including PD, Stroke, TBI and SCI. The potential to amplify these neuroplastic effects by combining exercise with neuromodulation is an exciting frontier in personalized rehabilitation. Until recently, the majority of studies have concentrated solely on the effects of neuromodulation, without integrating it with any specific behavioral, physical, or occupational therapy. Nevertheless, more research is emerging to unite these two therapeutic strategies to create a synergistic effect that could enhance patient outcomes (Bolognini et al., 2009). This emerging approach is founded on the hypothesis that motor learning through exercise and neuromodulation may have complementary mechanisms of action that lead to neuroplasticity in the human cortex. Concurrent employment of these adjuvant therapies could be more advantageous than using them independently (Bolognini et al., 2009).

After a comprehensive review, we have provided suggestions for future investigation of combined neuromodulation and physical rehabilitation to optimize outcomes based on the principles of neuroplasticity and the physiologic effects of various neuromodulatory mechanisms including TMS, PNS, and VNS. Additionally, we have provided a brief overview of additional neuromodulatory methods including tDCS and focused ultrasound. The overarching aim of this review is to serve as a resource for clinicians striving to combine neuromodulation with rehabilitation, as well as to encourage researchers to optimize these approaches for broad clinical application. We hope to propel advancements in personalized physical therapy, establishing initial guidelines for diagnosis-specific protocols, ultimately amplifying the effectiveness of rehabilitation outcomes. See Table 4 for a summary of potential research applications.

**TABLE 4:** Potential research applications for neuromodulation in rehabilitation.

Diagnosis	Neuromodulation technique	Suggested areas of research
Parkinson’s disease	TMS	rTMS over M1 prior to high amplitude training (LSVT BIG, PWR Moves) (Fox et al., 2012; Yang et al., 2018; Osborne et al., 2022)
	PNS	FES for tremor reduction combined with UE functional training (Popa and Taylor, 2015; Meng et al., 2022)
	VNS	VNS during most successful trials of high-amplitude training (LSVT BIG, PWR Moves) (Fox et al., 2012; Marano et al., 2022; Osborne et al., 2022)
Stroke	TMS	iTBS combined with UE CIMT for individuals with chronic stroke (Nesin et al., 2019; Gao et al., 2022)
	PNS	Afferent PNS combined with repetitive task practice (Fleming et al., 2015; Kim et al., 2020)
	VNS	VNS during most successful trials of shaping tasks during CIMT (Hays et al., 2014a,b; Dawson et al., 2016, 2021; Nesin et al., 2019)
TBI	TMS	rTMS prior to vestibular therapy in mild TBI93 (Schlemmer and Nicholson, 2022),
	PNS	Chronic TBI–FES to hemiparetic UE during repetitive task Practice (Ambrosini et al., 2011; Lee et al., 2019; Milosevic et al., 2021)
		Acute TBI–NMES combined with functional sit < > stand training for critically ill patients (Canning et al., 2003; Silva et al., 2019)
	VNS	Mild TBI–VNS for symptom management prior to vestibular Therapy (Pruitt et al., 2016; Hakon et al., 2020; Noé et al., 2020; Schlemmer and Nicholson, 2022)
		Severe TBI–VNS paired with successful trials during stimulation protocol for DOC Recovery (Pruitt et al., 2016; Thibaut et al., 2019; Hakon et al., 2020; Noé et al., 2020)
SCI	TMS	Concurrent rTMS with NMES during LE biking (FES bike) for incomplete SCI (Belci et al., 2004; van der Scheer et al., 2021)
	PNS	FES combined with UE and/or LE cycling for individuals with acute, chronic, complete and incomplete SCI (Mangold et al., 2005; Kapadia et al., 2011, 2014; van der Scheer et al., 2021)
	VNS	VNS applied with peak power output during FES UE/LE Cycling (Ganzer et al., 2018b; Darrow et al., 2020; van der Scheer et al., 2021)

## Author contributions

AE and WT contributed to conception and design of the review manuscript. AE wrote the first draft of the manuscript. WT and KM completed article review and provided suggested edits. All authors approved the submitted version.
